# Supplementary material for: Projected health impact of post-discharge malaria chemoprevention among children with severe malarial anaemia in Africa
Source: Nat Commun. 2023 Jan 25;14:402. doi: 10.1038/s41467-023-35939-w (PMC9876927; doi:10.1038/s41467-023-35939-w)
Supplement: Supplementary file 3 — Reporting Summary [file 41467_2023_35939_MOESM3_ESM.pdf]

nature portfolio

Corresponding author(s): Lucy Okell, Amani Thomas Mori

Last updated by author(s): Jul 18, 2022

Reporting Summary

Nature Portfolio wishes to improve the reproducibility of the work that we publish. This form provides structure for consistency and transparency in reporting. For further information on Nature Portfolio policies, see our Editorial Policies and the Editorial Policy Checklist.

Statistics

For all statistical analyses, confirm that the following items are present in the figure legend, table legend, main text, or Methods section.

n/a	Confirmed	
☐	☒	The exact sample size (*n*) for each experimental group/condition, given as a discrete number and unit of measurement
☐	☒	A statement on whether measurements were taken from distinct samples or whether the same sample was measured repeatedly
☒	☐	The statistical test(s) used AND whether they are one- or two-sided<br>*Only common tests should be described solely by name; describe more complex techniques in the Methods section.*
☐	☒	A description of all covariates tested
☐	☒	A description of any assumptions or corrections, such as tests of normality and adjustment for multiple comparisons
☐	☒	A full description of the statistical parameters including central tendency (e.g. means) or other basic estimates (e.g. regression coefficient) AND variation (e.g. standard deviation) or associated estimates of uncertainty (e.g. confidence intervals)
☒	☐	For null hypothesis testing, the test statistic (e.g. *F*, *t*, *r*) with confidence intervals, effect sizes, degrees of freedom and *P* value noted<br>*Give P values as exact values whenever suitable.*
☐	☒	For Bayesian analysis, information on the choice of priors and Markov chain Monte Carlo settings
☐	☒	For hierarchical and complex designs, identification of the appropriate level for tests and full reporting of outcomes
☒	☐	Estimates of effect sizes (e.g. Cohen's *d*, Pearson's *r*), indicating how they were calculated

Our web collection on statistics for biologists contains articles on many of the points above.

Software and code

Policy information about availability of computer code

Data collection	N/A this study did not collect new data.
Data analysis	Most analyses were conducted in R Version 4.2.0: R Core Team (2021). R: A language and environment for statistical computing. R Foundation for Statistical Computing, Vienna, Austria. URL https://www.R-project.org/.<br>Model fitting was undertaken using MCMC in the RStan software Version 2.21.2 (Supplementary reference 8).<br>A transmission model was used to generate inputs for the population analysis (Imperial College London; 'IC model', supplementary references 2,12,13).

For manuscripts utilizing custom algorithms or software that are central to the research but not yet described in published literature, software must be made available to editors and reviewers. We strongly encourage code deposition in a community repository (e.g. GitHub). See the Nature Portfolio guidelines for submitting code & software for further information.

Data

Policy information about availability of data

All manuscripts must include a data availability statement. This statement should provide the following information, where applicable:

- Accession codes, unique identifiers, or web links for publicly available datasets
- A description of any restrictions on data availability
- For clinical datasets or third party data, please ensure that the statement adheres to our policy

The parasite prevalence data are publicly available on the Malaria Atlas Project website https://malariaatlas.org/.23 Population estimates are available from WorldPop www.worldpop.org.43 The clinical trial data analysed during the current study will be made available when a research proposal has been approved by the investigators after consideration of overlap between the proposal and any ongoing efforts. Proposals should be directed to feiko.terkuile@lstmed.ac.uk and Bjarne.Robberstad@uib.no; to gain access, data requesters must sign a data access agreement, and the de-identified database will be transferred electronically.

Human research participants

Policy information about studies involving human research participants and Sex and Gender in Research.

Reporting on sex and gender	The dataset analysed includes all genders and sexes combined. We only had access to a de-identified minimal dataset which did not have access to sex or gender information and therefore analyses were not disaggregated in this way. In the original trial, placebo group study participants were 50.5% male, and the chemoprevention group was 53.4% male. There was no difference in intervention effect by sex (Kwambai et al 2020 NEJM).
Population characteristics	All participants were under 5 years of age.
Recruitment	In the original trial, 1125 children were assessed for eligibility; 1049 children underwent randomization and were included in the analysis presented here. This high retention rate minimises bias due to non responders etc. All participants were recruited in hospital, therefore the original study did not include participants who did not access hospital care. This is the subject of extensive sensitivity analysis in our paper.
Ethics oversight	The trials and analyses were approved by the ethics committees at the Kenya Medical Research Institute, Makerere University, the Western Norway Regional Committee for Medical and Health Research Ethics, the Liverpool School of Tropical Medicine, the University of Minnesota, and the Uganda National Council of Science and Technology.

Note that full information on the approval of the study protocol must also be provided in the manuscript.

Field-specific reporting

Please select the one below that is the best fit for your research. If you are not sure, read the appropriate sections before making your selection.

☒ Life sciences ☐ Behavioural & social sciences ☐ Ecological, evolutionary & environmental sciences

For a reference copy of the document with all sections, see nature.com/documents/nr-reporting-summary-flat.pdf

Life sciences study design

All studies must disclose on these points even when the disclosure is negative.

Sample size	N/A Secondary data analyses only (see Kwambai et al 2020 NEJM for sample size calculation during the original trial).
Data exclusions	We fit to incidence data from weeks 1-25 of follow up and excluded the final week of follow up (week 26) due to anomalous results. The recorded incidence of uncomplicated malaria was much higher in the last week of follow up than in any other week and was a clear outlier (Figure S7). This was driven by cases from the trial site in Jinja only.
Replication	Model predictions were compared against data from 3 further studies for validation (Figure 2B)
Randomization	The original trial which we analyse here was placebo-controlled and randomized. We perform secondary data analysis only.
Blinding	The original trial which we analyse here was double blind. We perform secondary data analysis only.

Reporting for specific materials, systems and methods

We require information from authors about some types of materials, experimental systems and methods used in many studies. Here, indicate whether each material, system or method listed is relevant to your study. If you are not sure if a list item applies to your research, read the appropriate section before selecting a response.

Materials & experimental systems

n/a	Involved in the study
☒	☐ Antibodies
☒	☐ Eukaryotic cell lines
☒	☐ Palaeontology and archaeology
☒	☐ Animals and other organisms
☐	☒ Clinical data
☒	☐ Dual use research of concern

Methods

n/a	Involved in the study
☒	☐ ChIP-seq
☒	☐ Flow cytometry
☒	☐ MRI-based neuroimaging

Clinical data

Policy information about clinical studies

All manuscripts should comply with the ICMJE guidelines for publication of clinical research and a completed CONSORT checklist must be included with all submissions.

Clinical trial registration	Secondary analysis of data only, but original trial was registered with ClinicalTrials.gov, NCT02671175 on 28 January 2016
Study protocol	Original data: protocol is here: Kwambai TK, Dhabangi A, Idro R, et al. Malaria chemoprevention with monthly dihydroartemisinin-piperaquine for the post-discharge management of severe anaemia in children aged less than 5 years in Uganda and Kenya: study protocol for a multi-centre, two-arm, randomised, placebo-controlled, superiority trial. Trials 2018; 19: 610.
Data collection	Data used for secondary analysis in this manuscript were collected from a previous trial of PDMC conducted between 2016-2018 in nine hospitals in areas with moderate-to-intense perennial malaria transmission in Kenya and Uganda. See also Kwambai et al 2020 NEJM.
Outcomes	Outcomes were hospitalized malaria episodes and uncomplicated malaria episodes. These were assessed in the study hospitals and clinics (see Kwambai et al 2020).
